# Preclinical Analysis of Fetal Human Mesencephalic Neural Progenitor Cell Lines: Characterization and Safety In Vitro and In Vivo

**DOI:** 10.5966/sctm.2015-0228

**Published:** 2016-09-02

**Authors:** Jisook Moon, Sigrid C. Schwarz, Hyun‐Seob Lee, Jun Mo Kang, Young‐Eun Lee, Bona Kim, Mi‐Young Sung, Günter Höglinger, Florian Wegner, Jin Su Kim, Hyung‐Min Chung, Sung Woon Chang, Kwang Yul Cha, Kwang‐Soo Kim, Johannes Schwarz

**Affiliations:** ^1^ Department of Biotechnology, College of Life Science, CHA University, Seongnam‐si, Gyeonggi‐do, Korea; ^2^ General Research Division, Korea Research‐Driven Hospital, Bundang CHA Medical Center, CHA University, Seongnam‐si, Gyeonggi‐do, Korea; ^3^ German Center for Neurodegenerative Diseases, Technical University Munich, Munich, Germany; ^4^ Development Division, CHA Biotech, Seongnam‐si, Gyeonggi‐do, Korea; ^5^ Department of Neurology, Hannover Medical School, Hannover, Germany; ^6^ Molecular Imaging Research Center, Korea Institute of Radiological and Medical Sciences, Seoul, Korea; ^7^ Department of Stem Cell Biology, Graduate School of Medicine, Konkuk University, Gwangjin‐gu, Seoul, Korea; ^8^ Department of Obstetrics and Gynecology, CHA Bundang Medical Center, CHA University, Seongnam‐si, Gyeonggi‐do, Korea; ^9^ Molecular Neurobiology Laboratory, Department of Psychiatry, Program in Neuroscience and Harvard Stem Cell Institute, McLean Hospital/Harvard Medical School, Belmont, Massachusetts, USA

**Keywords:** Human fetal midbrain tissue, Neural progenitor cells, Dopaminergic differentiation, Transplantation, Rodents, Preclinical safety, Parkinson's disease

## Abstract

We have developed a good manufacturing practice for long‐term cultivation of fetal human midbrain‐derived neural progenitor cells. The generation of human dopaminergic neurons may serve as a tool of either restorative cell therapies or cellular models, particularly as a reference for phenotyping region‐specific human neural stem cell lines such as human embryonic stem cells and human inducible pluripotent stem cells. We cultivated 3 different midbrain neural progenitor lines at 10, 12, and 14 weeks of gestation for more than a year and characterized them in great detail, as well as in comparison with Lund mesencephalic cells. The whole cultivation process of tissue preparation, cultivation, and cryopreservation was developed using strict serum‐free conditions and standardized operating protocols under clean‐room conditions. Long‐term‐cultivated midbrain‐derived neural progenitor cells retained stemness, midbrain fate specificity, and floorplate markers. The potential to differentiate into authentic A9‐specific dopaminergic neurons was markedly elevated after prolonged expansion, resulting in large quantities of functional dopaminergic neurons without genetic modification. In restorative cell therapeutic approaches, midbrain‐derived neural progenitor cells reversed impaired motor function in rodents, survived well, and did not exhibit tumor formation in immunodeficient nude mice in the short or long term (8 and 30 weeks, respectively). We conclude that midbrain‐derived neural progenitor cells are a promising source for human dopaminergic neurons and suitable for long‐term expansion under good manufacturing practice, thus opening the avenue for restorative clinical applications or robust cellular models such as high‐content or high‐throughput screening. Stem Cells Translational Medicine *2017;6:576–588*

**Significance Statement**

For many years, human fetal midbrain‐derived neural stem or progenitor cells have been considered a potential source for restorative therapy in patients with Parkinson's disease. However, there have been considerable concerns with respect to limited expansion, early senescence, and loss of pluripotency. This study investigated such cells thoroughly and developed techniques that allow for long‐term stable expansion, overcoming the above concerns. The cells were found to be safe and potent, allowing clinical application.

## Introduction

Cell therapy is a promising treatment for Parkinson's disease, although its clinical application with primary tissue is limited because of restricted resources and minimum effective dosage. Hence, neuroscientists are focusing on new restorative approaches with human embryonic stem cells (hESCs) and fetal cell‐based treatments (http://www.gforce-pd.com and http://www.transeuro.org.uk) 1, 2, 3, 4, 5, 6, 7, 8, 9. Along with hESCs 1, 6, 7 and genetically modified induced pluripotent stem cells (IPSCs) 2, 3, 8, 9, 10, human midbrain‐derived neural progenitor cells (hmNPCs) have the capacity to self‐renew and to differentiate into dopaminergic (DA) neurons 11, 12, 13, 14, 15, 16, 17. Here, we report that hmNPCs remain fate specific and differentiate into defined, authentic A9‐specific dopaminergic neurons. We developed a good manufacturing practice (GMP) for successful generation and characterization of unmodified, stable, functional human A9‐specific DA neurons from three different hmNPC lines in significant quantities after serum‐free, GMP‐compliant, long‐term cultivation.

Expression of hmNPC characteristic genes, proliferation markers, differentiation potential, and multipotency was analyzed in 3 different hmNPC lines over the initial 20 passages. We were able to show that there was no decline in growth rate. We detected an increase in expression of stem cell markers over time and also a significant increase in dopaminergic differentiation potential. hmNPCs retain floorplate markers and midbrain identity. Grafted hmNPCs significantly reduced motor asymmetry and survived intrastriatally in the 6‐hydroxydopamine (6‐OHDA)‐lesioned adult rat brain. Karyotyping, DNA repair arrays, and in vivo teratoma formation assays, performed under strict good laboratory practice (GLP) compliance, confirmed preclinical safety of hmNPCs.

## Methods

### Cell Cultivation

All experiments were approved by the ethics committee of the University of Leipzig and the Technical University of Munich, Germany, or CHA University/Hospital, Korea, and were in accordance with all state and federal guidelines.

hmNPCs were generated from central nervous system tissue of spontaneously aborted fetal human fetuses of female gender at gestational week (GW) 10, 12, and 14 with mother's consent. Fetal tissues were screened for relevant pathogens mentioned in Annex II of the EMEA Commission Directive 2006/17/EC, such as human immunodeficiency virus 1/2, hepatitis C, hepatitis B, *Treponema pallidum*, cytomegalovirus, and *Toxoplasma gondii*. In addition, endotoxin levels were evaluated. Only hmNPC lines from fetuses without any diagnostic findings were used for long‐term cultivation.

Briefly, human fetal tissue at GW 10, 12, and 14 was prepared with a time delay of 1–3 hours after termination of pregnancy. The brain tissue was washed with sterile Hanks balanced salt solution and dissected into mesencephalic and nonmesencephalic primary tissue samples. The tissues were mechanically separated into small pieces, incubated in 0.1 mg/ml Papain solution (Roche, Basel, Switzerland, http://www.roche.com) supplemented with 10 µg/ml DNase (Roche) for 30 minutes at 37°C, washed three times with Hanks balanced salt solution, and incubated in 50 µg/ml antipain solution (Roche) for 30 minutes at 37°C. After 3 further washing steps, samples were homogenized 20 times by gentle trituration. Propagation of the cell suspension was performed in Hanks balanced salt solution and cell culture flasks coated with poly‐l‐ornithine (15 µg/ml; Sigma Aldrich, St. Louis, MO, http://www.sigmaaldrich.com) and fibronectin (200 µl/ml; EMD Millipore, Darmstadt, Germany, http://www.emdmillipore.com). The expansion medium was based on Dulbecco's modified Eagle's medium/Ham's F12 (PAA Laboratories, Dartmouth, MA, http://www.gelifesciences.com) supplemented with 2% B27 supplement minus‐AO (2%, B‐27 Supplement XenoFree CTS; Thermo Fisher Scientific Life Sciences, Waltham, MA, http://www.thermofisher.com), human recombinant epidermal growth factor, and human recombinant fibroblast growth factor 2 (20 ng/ml; PeproTech, Rocky Hill, NJ, http://www.peprotech.com). Supplemented medium was changed every other day. A preparation time of ∼4 hours was needed from tissue preparation until cell seeding. Long‐term expansion of the cells (>6 months) was enabled in reduced atmospheric oxygen (2%–3%) 14, 15, 18. For passaging, cell detachment was induced by undiluted accutase solution (PAA Laboratories) for 30 minutes at 37°C at a confluence of 80%–100%.

For dopaminergic differentiation, cells were plated onto precoated cell culture dishes. After the cells reached 80%–100% confluence, the medium was exchanged for neurobasal medium (Thermo Fisher Scientific Life Sciences) containing additives such as GlutaMAX (1%; Thermo Fisher Scientific Life Sciences), B‐27 supplement minus‐AO (2%), forskolin (10 µM; Sigma‐Aldrich), picolinic acid derivative (50 µM, Sigma‐Aldrich), and dibutyryl cyclic adenosine monophosphate (100 µM; Sigma‐Aldrich). The cells were differentiated for 7 days. An immortalized cell line from fetal midbrain neural progenitor cells (Lund mesencephalic cells [LUHMES]) were cultivated as a reference cell line according to the literature 19.

### Karyotype and DNA Repair Analysis

Karyotyping was performed by the Cytogenomic Services Facility of Sumkwang, Medical Laboratories, Korea. To analyze karyotype, cell division was blocked in metaphase by 0.05 μg/ml colcemid for 1–2 hours. The chromosomes were visualized by G‐band staining, and >100 metaphase cells were analyzed 20. To evaluate the DNA repair and stability during long‐term expansion, cell lines at early (P5) and late (>P20) passages were analyzed with the PAHS‐042Z RT^2^ Profiler polymerase chain reaction (PCR) array (Qiagen, Hilden, Germany, http://www.qiagen.com) according to the manufacturer's instructions.

### Fluorescence‐Activated Cell Sorting

Undifferentiated and differentiated hmNPCs (1 × 10^5^ of each sample) were resuspended in 75 µl phosphate‐buffered saline (PBS; pH 7.4) and incubated with 0.1 µg of the primary antibody against CD15, CD133, and CD184 (supplemental online Table 5) for 60 minutes at 37°C. As negative controls, undifferentiated and differentiated hmNPCs without incubation in primary antibody were used. Cells were washed with 1 ml PBS and 1% heat inactivated fetal bovine serum. After resuspension in 300 µl PBS, samples were analyzed by flow cytometry using FACScan (BD, Franklin Lakes, NJ, http://www.bd.com). The sample flow rate during analysis did not exceed 300–400 cells per second.

### RNA Extraction, Reverse Transcription, and Semiquantitative Real‐Time PCR

For quantitative real‐time PCR analysis (qPCR), SYBR Green Select qPCR Supermix (Thermo Fisher Scientific Life Sciences), 5 ng complementary DNA from total RNA, 0.2 μM forward and reverse primers, and 100 nM 5‐carboxy‐X‐rhodamine (passive references dye) were used. Oligonucleotide primers (supplemental online Table 4) were designed using Primer3 software to flank intron sequences if possible. PCR was performed in a Step One Plus instrument (Thermo Fisher Scientific Life Sciences) using the following protocol: 2 minutes at 50°C, 2 minutes at 95°C, and 40 cycles of 15 seconds at 95°C and 60 seconds at 60°C. Melting curves were recorded. The correct size of the respective single amplicons was assured by agarose gel electrophoresis, and the identity was verified by restriction enzyme cleavage or sequencing for at least 10% of the amplicons. Cycle threshold (CT) values were set within the exponential phase of the PCR. Data were normalized to five housekeeping genes, *TBP*, *GPBP1*, *PSMC1*, *UBQLN2*, and *RPL22*, and either ΔCT values were used to calculate the relative expression levels or the comparative normalized relative quantity (CNRQ) was used. Gene regulation was statistically evaluated by subjecting the ΔΔCT values or CNRQ to a two‐tailed Student's *t* test, assuming equal variances.

### Immunocytochemistry

Cells were fixed with 4% paraformaldehyde. Fixed cells were permeabilized with 0.2% Triton X‐100. Unspecific binding was blocked in PBS supplemented with 2% bovine serum albumin and 3% chicken or donkey serum. Incubation followed with primary antibodies overnight at 4°C in blocking buffer. The primary antibodies are summarized in supplemental online Table 5. After washing, the cells were incubated with fluorescent secondary antibodies Alexa Fluor 488 conjugate or Alexa Fluor 594 conjugate (1:500; Thermo Fisher Scientific Life Sciences) for 1 hour at room temperature. Nuclei were stained with 4′,6‐diamidino‐2‐phenylindole (DAPI; 0.5 mg/ml; EMD Millipore) for 5 minutes at room temperature.

Coverslips were mounted onto glass slides and examined under a fluorescence microscope (Axiovert 200; Zeiss, Oberkochen, Germany, http://www.zeiss.com). Digital images were acquired with the AxioCam MRc camera using image‐analysis software AxioVision 4 (Zeiss). The percentage of labeled cells was determined by counting the number of positive cells in relation to the number of DAPI‐stained nuclei. Approximately 2,000–3,000 cells were counted within 6 randomly selected fields per well in a single‐blinded fashion by the German and Korean research teams. Neurite length was measured in a single‐blinded fashion using a Leica confocal microscope (Leica TCSSP5x, Leica Application Suite Software). Immunohistochemistry of postmortem brains was performed as previously described 4, 21 with the antibodies described in supplemental online Table 5.

### Quantitative Determination of Dopamine Release Using Enzyme‐Linked Immunosorbent Assay

The concentration of dopamine released from cultured hmNPCs (undifferentiated versus differentiated, *n* = 3) was determined using a dopamine enzyme‐linked immunosorbent assay (ELISA) kit according to the manufacturer's instructions (IBL International, Morrisville, NC, http://www/ibl-international.com). As a positive control, DA release of PC12 cells was tested (data not shown).

### In Vivo Transplantation Experiments

#### Rodents

Female adult Sprague‐Dawley rats (220–250 g, ∼10 weeks of age; Charles River Laboratories, Wilmington, MA, http://www.criver.com) were used in this study. The experimental procedure was carried out according to the animal care guidelines of the Institutional Animal Care and Use Committees in Germany and Korea.

#### 6‐OHDA Lesions and Transplantation

Rats (*n* = 18 per group) were given 6‐OHDA as specified 22. Four weeks after lesion induction, rats were tested for motor asymmetry as described 23. Rats with at least six ipsilateral turns/minute were randomly divided into three groups: sham controls and graft recipients of undifferentiated or differentiated hmNPCs. On transplantation day, cell vitality before and after grafting was more than 90% (undifferentiated cells, 91.2% ± 0.94%; differentiated cells, 93.3% ± 0.49%). Cell suspension (3 µl of 1.5 × 10^5^ cells per μl in PBS) was injected into the lesioned striatum using a KDS310 nano pump (KD Scientific, Holliston, MA, http://www.kdscientific.com).

### Positron Emission Tomography Analysis

The Inveon positron emission tomography (PET) scanner (Siemens Medical Solutions, Knoxville, TN, http://usa.healthcare.siemens.com) was used in the present analysis 24. Dopaminergic impairment and effect of transplantation of hmNPCs were measured using [^18^F]*N*‐3‐fluoropropyl‐2β‐carboxymethoxy‐3β‐(4‐iodophenyl)‐nortropane ([^18^F]FP‐CIT) PET as described 25. Nine weeks after transplantation, two rats (sham and hmNPC grafted) that showed average rotation numbers were selected and underwent [^18^F]FP‐CIT PET scans with 1 mCi of [^18^F]FP‐CIT. One‐hour emission data were acquired with an energy window of 350–650 KeV and a coincidence window of 3.432 nanoseconds. Emission listmode data were sorted into a 3D sinogram and reconstructed using a 3D rapid prototyping reconstruction algorithm 26 (matrix size 256 × 256 × 159). Attenuation and scatter corrections for the emission PET data were performed. Image pixel sizes were 0.0017 mm transaxially and 0.0079 mm axially. To delineate regions of interest, the maximum a posteriori probability algorithm 27 was also used for image reconstruction.

### Behavioral Analysis

Rotation behavior test by amphetamine (3 mg/kg intraperitoneally; Sigma‐Aldrich) was measured for 90 minutes in automatized Rotameter bowls (Med Associates, St. Albans City, VT, http://www.med-associates.com). Testing was performed 3, 6, 9, and 12 weeks after transplantation.

### Teratoma Formation Assay

The teratoma formation assay was performed according to the literature 28, 29, 30. For transplantation, hmNPCs (P12), expanded to >80% confluence, were harvested and dissociated into cell suspensions. The vitality of hmNPCs was >90% before transplantation. BALB/c‐ν Sic male mice (6 weeks old; Central Laboratory Animal, Seoul, Korea, http://www.labanimal.co.kr) were transplanted with 5 × 10^5^ undifferentiated hmNPCs, differentiated hmNPCs, or tumor cells (positive control) in 20 µl PBS into the right testis. The left testis was used as sham control. Eight (*n* = 5) or 30 (*n* = 5) weeks after transplantation, mice were sacrificed, and each testis was fixed immediately in 10% buffered formaldehyde. All tissues were embedded in a paraffin block for histological analysis. The blocks were sectioned (10 µm thickness), stained with hematoxylin and eosin, and tested for nestin (immunocytochemistry [ICC]) and human chromosome 17 (fluorescent in situ hybridization [FISH]). Nestin‐immunoreactive (IR) cells were counted in sham control and hmNPC‐grafted testicles. To compensate for double counting in adjacent sections, Abercrombie's correction was used.

### FISH Method

Detection of aneuploidies for human chromosome 17A with one‐color FISH assay was performed (Spectrum Orange, 431105; Abbott Molecular, Des Plaines, IL, https://www.abbottmolecular.com) in mouse testis samples (*n* = 3) 30 weeks after hmNPC injection. Tissues were fixed in paraformaldehyde, embedded in paraffin, dewaxed, and rehydrated. After washing in saline‐sodium citrate (SSC) buffer at room temperature for 30 minutes, a mixture of probes was applied to each slide. The probes were denatured at 75°C for 5 minutes and then hybridized at 37°C overnight. After washing in 2× SSC at 60°C for 2 minutes, cells were counterstained with DAPI.

### Statistical Analysis

Statistical analyses were conducted on a CHA University mainframe computer using the Statistical Analysis System (SAS; SAS Korea, Seoul, Korea, http://www.sas.com), version Enterprise 4.0. For a behavioral assay, mean scores from the Rotation Test were used as the dependent variables, and treatment (sham control and undifferentiated and differentiated hmNPC transplantation groups) and time (1 week pretransplantation; 3, 6, 9, and 12 weeks post‐transplantation) were used as independent variables. Most of the statistical analysis was performed using analysis of variance or a mixed model analysis of variance procedure (SAS, PROC MIXED) with Fisher's least significant difference (LSD) post hoc tests to account for random effects. The number of injected cells was analyzed using one‐ or two‐way analysis of variance followed by LSD post hoc tests. An independent Student's *t* test was used for two‐group analysis. Results are expressed as means ± SEM. In all analyses, differences were considered significant at *p* < .05.

## Results

### Efficient Long‐Term Propagation of hmNPC Lines

**Figure 1:**
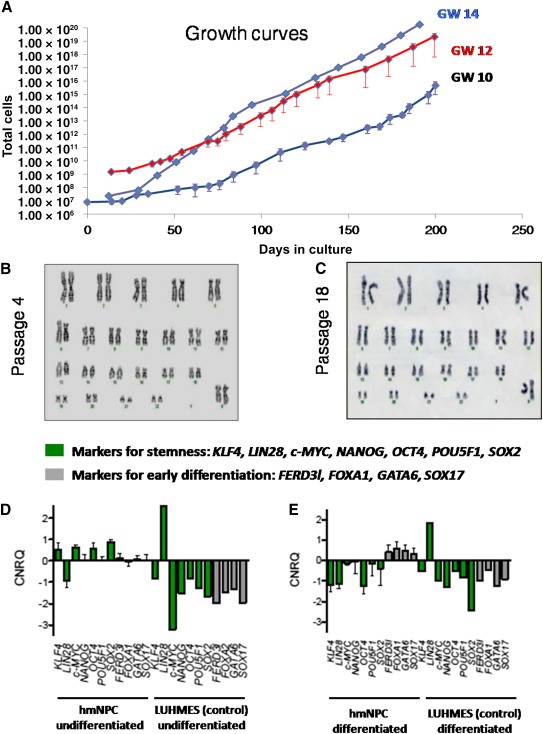
Long‐term propagation of hmNPC lines. **(A):** Growth curves of hmNPCs at GW 10, 12, and 14 (mean ± SEM). hmNPCs of GW 14 display the highest growth rate. **(B):** Karyotyping of GW 12 hmNPCs at early passage established no chromosomal abnormalities. **(C):** Karyotyping of GW 12 hmNPCs at late passage; no chromosomal abnormalities were found. **(D):** Gene expression in undifferentiated hmNPCs and undifferentiated immortalized LUHMES cells. All expression levels were calibrated and normalized relative to quantity with respective standard curves (CNRQ). Green, pluripotency markers; gray, markers involved in early differentiation. Data are presented as mean ± SEM, *n* = 3 hmNPC, *n* = 1 LUHMES. *KLF4*, *c‐MYC*, *OCT4*, and *SOX2* were significantly upregulated in hmNPCs compared with differentiated hmNPCs and LUHMES cells (undifferentiated and differentiated). Genes involved in early differentiation such as *FERD3l*, *FOXA1*, *GATA6*, and *SOX17* were expressed at a low level. **(E):** Gene expression in differentiated hmNPCs and differentiated immortalized LUHMES cells. All expression levels were calibrated and normalized relative to quantity with respective standard curves (CNRQ). Green, pluripotency markers; gray, markers involved in early differentiation. The data are presented as mean ± SEM, *n* = 3 hmNPC, *n* = 1 LUHMES. Genes involved in stemness such as *KLF4*, *c‐MYC*, *OCT4*, and *SOX2* were significantly downregulated in differentiated hmNPCs compared with undifferentiated hmNPCs **(D)** and LUHMES cells (undifferentiated and differentiated). Genes involved in early differentiation such as *FERD3L*, *FOXA1*, *GATA6*, and *SOX17* were expressed at a higher level in differentiated hmNPCs compared with undifferentiated hmNPCs and differentiated LUHMES cells. Abbreviations: CNRQ, comparative normalized relative quantity; GW, gestational week; hmNPC, human midbrain‐derived neural progenitor cell.

Growth curves of hmNPCs from three different donors and various gestational weeks displayed exponential growth over the entire observation period (Fig. 1A). hmNPCs at GW 14 proliferated more efficiently than those at GW 10 and 12. All cell lines exhibited a very consistent morphology over time (data not shown). To address chromosomal stability, karyotyping was performed throughout long‐term cultivation. Major chromosomal abnormalities, such as trisomies, were not detected (Fig. 1B, 1C); however, small alterations may not be displayed using this analysis. In addition, PCR arrays for DNA repair were used in cell lines of early (P5) and late (>P20) passages to detect changes in accordance with longstanding propagation. The expression of 84 key genes involved in base excision, nucleotide excision, mismatch, double‐strand break, and other repair processes of damaged DNA revealed no significant differences between early and late passages of hmNPCs (data not shown).

We developed a serum‐free expansion protocol under normoxia (3% O_2_) with poly‐l‐ornithine and fibronectin as an extracellular matrix. Stemness and early differentiation potential were studied in undifferentiated and differentiated hmNPCs in comparison with LUHMES cells, an immortalized clonal neuronal cell line derived from fetal human midbrain (Fig. 1D, 1E). Undifferentiated hmNPCs showed higher levels of expression of pluripotency‐associated genes such as *KLF4*, *LIN28*, *cMYC*, *NANOG*, *OCT4*, *POU5F1*, and *SOX2* compared with differentiated hmNPCs and LUHMES cells, with the exception of *LIN28*. These markers were expressed on a constant level in undifferentiated hmNPCs throughout long‐term cultivation, as shown for *SOX2* (supplemental online Fig. 2A) as an example. Markers for early differentiation FERD3l, FOXA1, GATA6, and SOX17 were expressed at higher levels in differentiated hmNPCs compared with undifferentiated hmNPCs. In comparison with immortalized LUHMES cells (undifferentiated and differentiated), the expression levels of early differentiation markers were higher in hmNPCs (undifferentiated and differentiated) (Fig. 1D, 1E).

**Figure 2:**
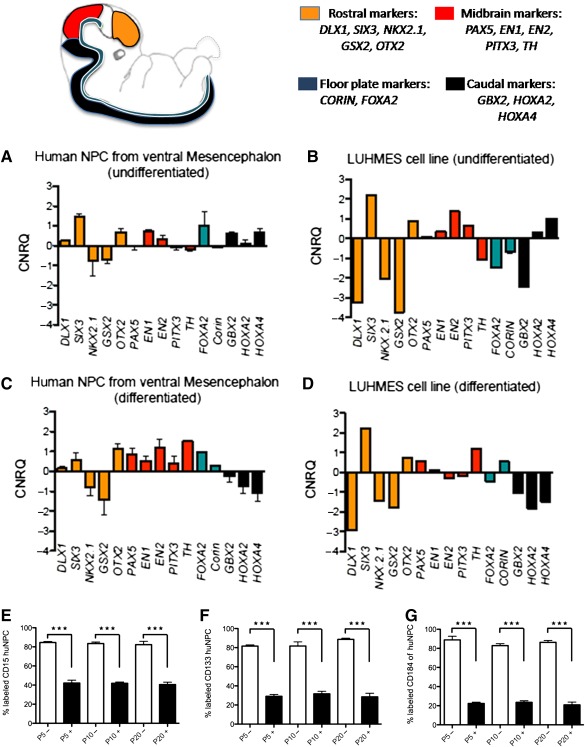
Phenotyping of hmNPCs. Gene expression analysis with markers for regional identity. Undifferentiated hmNPCs **(A)**, undifferentiated LUHMES cells **(B)**, differentiated hmNPCs **(C)**, and differentiated LUHMES cells **(D)** confirm midbrain identity of all three hmNPC lines and LUHMES cells. All expression levels were calibrated and normalized relative to quantity with respective standard curves (CNRQ). Orange, rostral markers; red, mesencephalic markers; green, ventral floorplate; black, caudal markers. Data are presented as mean ± SEM, *n* = 3 hmNPC, *n* = 1 LUHMES. Undifferentiated hmNPCs **(A)** and LUHMES cells **(B)** show expression of forebrain‐specific genes *SIX3* and *OTX2*. Genes involved in midbrain identity and the development of dopaminergic neurons such as *EN1* and *EN2* were markedly expressed. In addition, *PITX3* was present in LUHMES cells, whereas the floorplate marker *FOXA2* was expressed in hmNPCs. Caudal genes such as *GBX2*, *HOXA2*, and *HOXA4* were also present in undifferentiated hmNPCs. Differentiated hmNPCs expressed SIX3, OTX3, PAX5, EN1, EN2, PITX3, TH, FOXA2, and corin, whereas OTX2, PAX4, EN1, TH, and corin were present in LUHMES cells. FACS analysis with CD15 **(E)**, CD133 **(F)**, and CD184 **(G)** confirms that undifferentiated hmNPCs at passages 5, 10, and 20 (–P5, –P10, and –P20) display robust expression of proteins characteristic for neural progenitor cells such as CD15, CD133, and CD184 in long‐term propagation (white columns). After 7 days of differentiation, hmNPCs at passages 5, 10, and 20 (+P5, +P10, and +P20, black columns) show highly significant reductions of CD15‐, CD133‐, and CD184‐positive cells (∗∗∗, *p* < .0001). Abbreviations: CNRQ, comparative normalized relative quantity; FACS, fluorescence‐activated cell sorting; GW, gestational week; hmNPC, human midbrain‐derived neural progenitor cell.

To confirm tissue specificity, the collected hmNPC lines were screened for TH (supplemental online Fig. 1A) and regionalized expression patterns with marker genes specific for forebrain, midbrain, hindbrain, and floorplate 1. The hmNPC lines displayed an expression profile of *PAX5*, *EN1*, *EN2*, *PITX3*, and *TH* consistent with midbrain identity 1. In line with the expression pattern of rostral and caudal markers, we also established the expression of floorplate markers such as *FOXA2* and *CORIN* in long‐term‐cultivated hmNPCs (Fig. 2A). Again, as a positive control, we used immortalized LUHMES cells before and after differentiation (Fig. 2B, 2D). The expression pattern of hmNPCs was comparable to that of LUHMES cells; however, differentiated hmNPCs showed a higher level of expression of midbrain markers (Fig. 2C). In addition, undifferentiated human neural progenitor cell lines derived from the forebrain (supplemental online Fig. 1B), as well as the hindbrain with metencephalon and spinal cord (supplemental online Fig. 1C), were screened for rostral and caudal markers. It is noteworthy that the described hindbrain markers *HOXA2* and *HOXA4* were expressed at a higher level in undifferentiated hmNPCs compared with human hindbrain areas such as metencephalon and spinal cord.

To characterize neural stem cells, we used CD15, CD133, and CD184 31, 32, 33, 34, 35, which are often referred to as characteristic markers of immature neural progenitor cells 31, 32, 33, 34, 35. Fluorescence‐activated cell sorting (FACS) analysis revealed that >80% of undifferentiated hmNPCs in the present study expressed these three proteins, without any effect of donor age or passage number (Fig. 2E–2G). The analyzed cell lines also showed a significant difference between undifferentiated and differentiated hmNPCs over time for CD15 [Fig. 2E; *F*(1,36) = 580.98, *p* < .0001], CD133 [Fig. 2F; *F*(1,36) = 677.61, *p* < .0001], and CD184 [Fig. 2G; *F*(1,36) = 1,211.74, *p* < .0001], indicating that a significant proportion of hmNPCs began differentiation after 7 days.

### Increased Dopaminergic Potential of hmNPCs in Long‐Term Cultivation

To explore the dopaminergic potential of all three hmNPC lines, we compared undifferentiated and differentiated hmNPCs at different passages (P5, P10, and P20) using qPCR, ICC, and ELISA for dopamine content.

**Figure 3:**
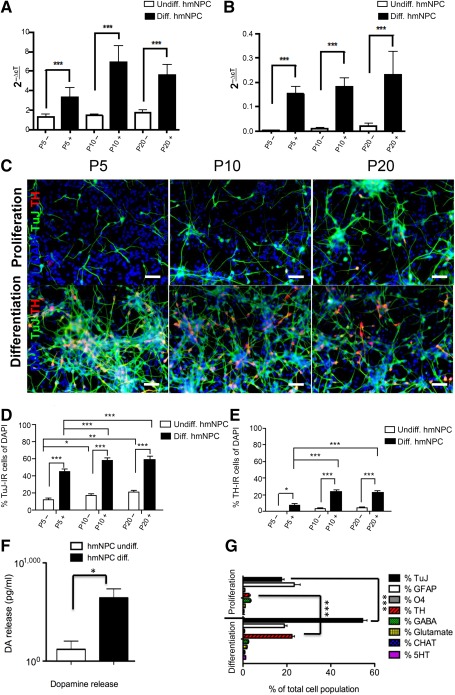
Dopaminergic phenotype of hmNPCs. **(A):** Quantitative polymerase chain reaction analysis (qPCR) of *TUBB3*. Gene expression analyses in undifferentiated (undiff.; P5–, P10–, P20–, white column) and differentiated (diff.; P5+, P10+, P20+, black column) hmNPC lines during long‐term cultivation for *TUBB3* transcription levels were significantly higher in differentiated hmNPCs compared with undifferentiated hmNPCs (*n* = 3, *p* < .0001, 2^−∆ct^ values) (supplemental online Tables 1, 2). **(B):** qPCR of *TH*. In parallel, hmNPC lines displayed a significant increase of expression levels of *TH* in differentiated hmNPCs (*p* < .0001, 2^−∆ct^ values) (supplemental online Tables 1, 2) compared with undifferentiated hmNPCs. **(C):** Photomicroplates with immunofluorescence for TuJ and TH in undifferentiated and differentiated hmNPCs (GW 10, 12, and 14) in early (P5), middle (P10), and late passages (P20) show a significant increase of TuJ and TH in undifferentiated hmNPCs over time. Scale bar = 100 µm. **(D):** Immunocytochemistry (ICC) quantification of TuJ‐IR hmNPC. ICC analysis revealed that the increase of TuJ in long‐term cultivation of differentiated hmNPCs was markedly significant: ∗, *p* < .05; ∗∗, *p* < .01; ∗∗∗, *p* < .0001. **(E):** ICC quantification of TH‐IR hmNPC. In parallel, ICC analysis showed a significant increase of TH in long‐term cultivation of differentiated hmNPCs: ∗, *p* < .05; ∗∗, *p* < .01; ∗∗∗, *p* < .0001. **(F):** Dopamine release was markedly higher in differentiated hmNPCs (643.53 ± 91.52 pg/ml) compared with undifferentiated hmNPCs (120.6 ± 84.1 pg/ml; ∗, *p* < .01). **(G):** ICC quantification of different neural phenotypes such as TuJ, GFAP, O4, TH, GABA, glutamate, CHAT, and 5HT. During all three readout points of long‐term cultivation (P5, P10, and P20), the average percentage of TuJ to DAPI (54.43 ± 2.00) and the percentage of TH to DAPI (22.27 ± 1.14) in differentiated hmNPCs compared with the percentage of TuJ to DAPI (17.57 ± 1.18) and the percentage of TH to DAPI (2.80 ± 0.59) in undifferentiated hmNPCs was highly significant (∗∗∗, *p* < .0001). The amount of other neuronal phenotypes was less than 2.5% (Table 1). Abbreviations: CNRQ, comparative normalized relative quantity; DAPI, 4′,6‐diamidino‐2‐phenylindole; GW, gestational week; hmNPC, human midbrain‐derived neural progenitor cell.

*TUBB3* transcription levels were significantly higher in differentiated hmNPCs compared with undifferentiated hmNPCs [Fig. 3A; *n* = 3, *F*(1,18) = 34.46, *p* < .0001, 2^−∆ct^ values] (supplemental online Tables 1, 2). In addition, hmNPCs showed a significant increase of expression levels of *TH* in differentiated hmNPCs [Fig. 3B; *F*(1,18) = 34.46, *p* < .0001, 2^−∆ct^ values] (supplemental online Tables 1, 2) compared with undifferentiated hmNPCs.

ICC analysis of hmNPCs for the neuronal and dopaminergic markers TuJ and TH, respectively (Fig. 3C), established an average number of TuJ‐IR neurons of 17.3% ± 1.73% in undifferentiated hmNPCs (supplemental online Table 3) and 54.4% ± 2.0% (P5, P10, and P20; Table 1) in differentiated hmNPCs. The average percentage of TH‐IR neurons was 2.80% ± 0.59% in undifferentiated hmNPCs (supplemental online Table 3) and 22.3% ± 1.1% in differentiated hmNPCs (P5, P10, and P20; Table 1). The detailed numbers for early, middle, and late passages are displayed in supplemental online Table 3 (undifferentiated hmNPCs) and Table 2 (differentiated hmNPCs), showing a constant and significant increase of neuronal cells with dopaminergic phenotype over time [Fig. 3D; *F*(1,90) = 348.18, *p* < .0001] and TH‐IR cells with increasing passages [Fig. 3E; *F*(2,81) = 11.18, *p* < .0001].

**Table 1:** ICC analysis of differentiated hmNPCs in long‐term cultivation: immunoreactive cells at passages 5, 10, and 20

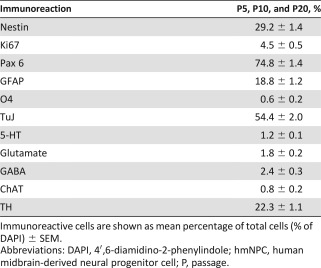

**Table 2:** ICC analysis for TuJ and TH in differentiated hmNPCs at passages 5, 10, and 20

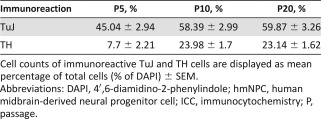

For complete phenotyping with ICC, other markers for proliferation and neural and neuronal cell types were analyzed (such as Ki67, SOX2, PAX6, GFAP, and O4) in undifferentiated hmNPCs (supplemental online Table 3). We found ∼20% of proliferating Ki67‐IR cells in long‐term‐cultivated hmNPCs and ∼90% of SOX2 and PAX6‐IR cells. The GFAP‐IR hmNPC population was on average 23.4% ± 4.9% (P5, P10, and P20), and the percentage of O4‐IR oligodendrocytes was 0.9% ± 0.2% (P5, P10, and P20). In addition to those markers, we studied other neuronal phenotypes in differentiated hmNPCs in long‐term cultivation such as serotonergic, cholinergic, GABAergic, and glutaminergic neurons (Table 1) and found only a small proportion (1%–2%) of other neuronal phenotypes (Fig. 3G). It is noteworthy that TH‐IR cells coexpressed GIRK2 (data not shown).

Immunoblots of all three long‐term‐cultivated hmNPC lines confirmed that TH and TuJ are expressed in increasing levels in undifferentiated and differentiated hmNPCs. The floorplate marker corin was also expressed in undifferentiated and differentiated hmNPCs (supplemental online Fig. 2C; supplemental online data).

The release of dopamine is a parameter for function of DA neurons and may thus be used as a potency assay. When stimulated with potassium, the differentiated hmNPCs exhibited a markedly higher DA release (643.53 ± 91.52 pg/ml) in contrast to undifferentiated hmNPCs (Fig. 3F; 120.6 ± 84.1 pg/ml, *p* < .01). As a positive control, PC12 cells were used (data not shown).

These data reveal that stemness and proliferation potential are preserved during prolonged proliferation of hmNPCs harvested at gestational weeks 10, 12, and 14. The generated hmNPC lines retained regional identity by expression of floorplate‐specific (*FOXA2* and *CORIN*) and midbrain‐specific (*PAX5*, *EN1*, *EN2*, *PITX3*, *TH*) markers during long‐term cultivation. Fetal hmNPCs of various gestational ages (GW 10, 12, and 14) offer great potential to differentiate into dopaminergic neurons during at least the initial 20 passages.

### Xenotransplanted hmNPCs Survive and Form Tumor‐Free Grafts in the Adult Rat Brain and in Immunodeficient Nude Mice

To address function and survival of the characterized hmNPCs, we grafted 2 × 10^5^ undifferentiated or differentiated hmNPCs into unilaterally lesioned 6‐OHDA rats, which were kept under immunosuppression with cyclosporine A for 12 weeks. Before intrastriatal transplantation, all graft recipients showed an average rotational asymmetry of 8.3 ± 0.5 (mean net ipsilateral turns per minute ± SEM). In the controls with sham injection, the motor asymmetry increased over time to 15.0 ± 1.7 at 12 weeks after surgery.

**Figure 4:**
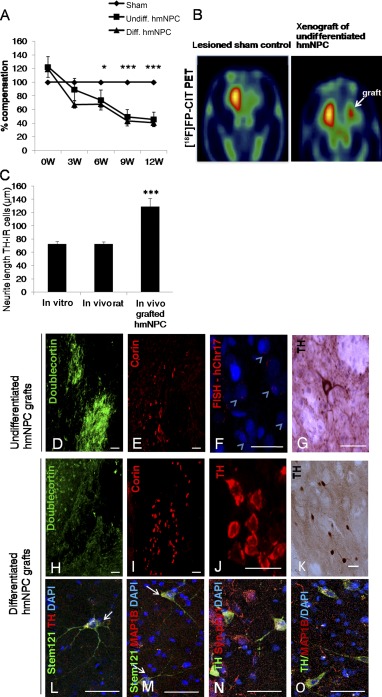
Xenogenic transplantation into unilaterally lesioned 6‐OHDA rats. **(A):** Percentage compensation of graft recipients with undifferentiated (undiff.) and differentiated (diff.) hmNPCs compared with sham controls showed a significant decrease of motor asymmetry 6, 9, and 12 weeks after grafting (∗, *p* < .05; ∗∗∗, *p* < .0001). W, weeks. **(B):** Functional imaging with PET showed distinct [^18^F]FP‐CIT binding in the transplantation area of grafted animal (right). No signal was found in the DA‐depleted striatum (STR) of sham‐injected control animal (left). **(C):** Quantification of neurite length of differentiated TH‐IR hmNPCs cultivated in vitro, intrinsic rat dopaminergic neurons of the substantia nigra (SN), and differentiated TH‐IR hmNPCs of intrastriatal grafts. Neurites of differentiated transplanted TH‐IR hmNPCs were significantly longer compared with differentiated hmNPCs in vitro and intrinsic dopaminergic rat TH‐IR neurons (∗∗∗, *p* < .0001). Doublecortin **(D)** and corin **(E)** staining of undifferentiated hmNPCs transplanted into the 6‐OHDA‐lesioned striatum measured 3 weeks after transplantation (scale bar = 50 µm). **(F):** FISH for human chromosome 17 (hChr.17) of undifferentiated hmNPCs transplanted into the 6‐OHDA‐lesioned striatum 12 weeks after grafting (scale bar = 20 µm). **(G):** TH staining of undifferentiated hmNPCs transplanted into the 6‐OHDA‐lesioned striatum 12 weeks after grafting (scale bar = 10 µm). Doublecortin **(H)** and corin **(I)** staining of differentiated hmNPCs transplanted into the 6‐OHDA‐lesioned striatum measured 3 weeks after transplantation (scale bar = 50 µm). TH **(J)** and TH‐DAB **(K)** staining of differentiated hmNPCs transplanted into the 6‐OHDA‐lesioned striatum 12 weeks after grafting (scale bar = 20 µm). STEM121 and TH **(L)**, STEM121 and MAP1B **(M)**, TH and synaptophysin 1 **(N)**, and TH and MAP1B **(O)** double staining of differentiated hmNPCs transplanted into the 6‐OHDA‐lesioned striatum 12 weeks after grafting (scale bar = 20 µm). Abbreviations: 6‐OHDA, 6‐hydroxydopamine; DA, dopaminergic; FISH, fluorescent in situ hybridization; FP‐CIT, *N*‐3‐fluoropropyl‐2β‐carboxymethoxy‐3β‐(4‐iodophenyl)‐nortropane; hmNPC, human midbrain‐derived neural progenitor cell; ICC, immunocytochemistry; IR, immunoreactive; PET, positron emission tomography.

Specific contrasts revealed that motor asymmetry was significantly compensated in graft recipients of undifferentiated (*p* = .36, *p* < .002, and *p* < .0001) and differentiated (*p* < .04, *p* < .0001, and *p* < .0001, respectively) hmNPCs compared with sham controls 6, 9, and 12 weeks after grafting. There was no significant difference in motor compensation between graft recipients of undifferentiated and differentiated hmNPCs (Fig. 4A). There was a statistically significant interaction between treatment and time in the rotation test [F(8,107) = 7.41, *p* < .001; Fig. 4A].

Functional ligand binding was tested in vivo by [^18^F]FP‐CIT PET 9 weeks after transplantation. The control as well as graft recipient showed normal [^18^F]FP‐CIT binding in the unlesioned striatum. No ligand binding was found in the DA‐depleted striatum of the sham control. The graft recipient showed distinct [^18^F]FP‐CIT binding in the transplantation area of the lesioned striatum (Fig. 4B).

Three and 12 weeks after surgery, animals were sacrificed. We found human neurons positive for doublecortin and corin at 3 weeks after transplantation, TH, and FISH hCHr17 at 12 weeks after transplantation in graft recipients of undifferentiated (Fig. 4D–4G) and differentiated (Fig. 4H–4O) hmNPCs. The morphology of the grafted hmNPCs was mostly round with neurite outgrowth. STEM121, the human‐specific cytoplasmic marker, was coexpressed with TH and MAP1B, a marker specific for developing neuronal cells, showing neurite outgrowth. TH‐IR neurons showed coexpression with synaptophysin 1, a presynaptic marker pointing toward neuronal integration. In addition, human nuclei‐IR cells were found in the transplantation area of grafted animals (supplemental online Fig. 3).

When comparing neurites of differentiated TH‐IR hmNPCs in a single‐blinded setting in vitro, in vivo, and with intrinsic rat TH‐IR neurons located in the SN, we found significantly longer neurites in transplanted differentiated hmNPCs (Fig. 4C; 129.14 ± 12.34 µm) compared with intrinsic rat TH‐IR neurites (72.49 ± 3.51 µm) and neurites of differentiated hmNPCs in vitro that were TH‐IR [72.39 ± 4.02 µm); F(1,72) = 19.06, *p* < .0001] after a survival time of 12 weeks.

**Figure 5:**
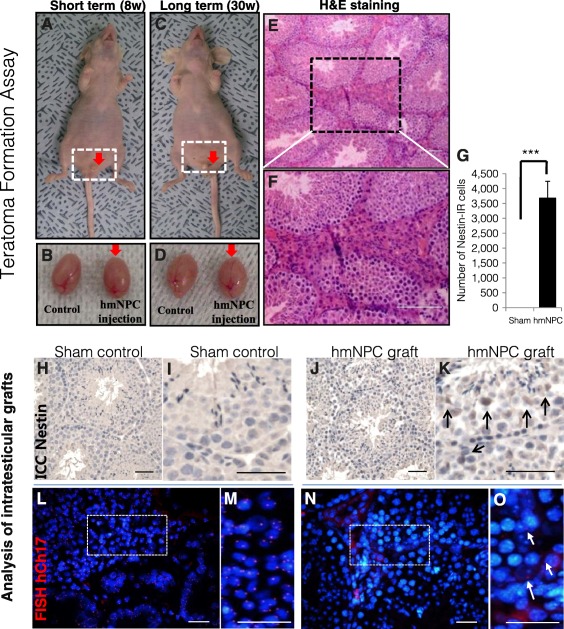
Safety. **(A):** Risk assessment of tumor potential for undifferentiated hmNPCs grafted into the left testicles of immunodeficient nude mice; 8 weeks survival time. w, weeks. **(B):** The right testicles served as sham control compared with the hmNPC grafts into the left testicles. No change of size and weight was observed between the testicles. **(C):** Risk assessment of tumor potential for undifferentiated hmNPCs grafted into the left testicles of immunodeficient nude mice. 30 weeks survival time. **(D):** The right testicles served as sham controls compared with the hmNPC grafts into the left testicles. No change of size and weight was observed between the testicles. Hematoxylin and eosin staining revealed no indication of tumor growth after 30 weeks: overview (scale bar = 100 μm) **(E)** and higher magnification (scale bar = 100 μm) **(F)**. **(G):** ICC quantification for nestin‐IR grafted cells. No nestin‐IR cells were detected in right sham control testicles. Photomicroplate showing no nestin‐IR cells in control testicle (scale bar = 50 µm) **(H)** and at higher magnification (scale bar = 100 µm) **(I)**. The hmNPC‐injected testis showed nestin‐IR cells measured at 30 weeks after hmNPC injection (scale bar = 100 µm) **(J)** and at higher magnification (scale bar = 100 µm; arrows indicate nestin‐IR cells) **(K)**. **(L):** FISH for human chromosome 17 (hCh17) was used to confirm intratesticular hmNPCs. Human lung cancer cells were used as positive control, showing 100% of hCh17‐positive cells (scale bar = 50 µm) **(L)** and at higher magnification (scale bar = 50 µm) **(M)**. FISH‐confirmed hCh17‐positive intratesticular hmNPC 30 weeks after transplantation (scale bar = 50 µm) **(N)** and at higher magnification (scale bar = 50 µm) **(O)**. Abbreviations: FISH, fluorescent in situ hybridization; H&E, hematoxylin and eosin; hmNPC, human midbrain‐derived neural progenitor cell; IR, immunoreactive.

For safety testing, undifferentiated and differentiated hmNPCs were grafted into testicles of immunodeficient nude mice for evaluation of toxicity and tumor potential. The GLP‐compliant toxicology screen revealed no indication of adverse effects (data not shown). After survival times of 8 and 30 weeks, animals were sacrificed and testicles were processed for postmortem analysis. HE staining revealed no indication for tumor formation of hmNPCs after 8 and 30 weeks (Fig. 5A–5F). There were significantly more nestin‐IR cells in the left testicles that received hmNPC grafts (Fig. 5G; *p* < .0001) compared with sham control (right testicles). The presence of long‐term‐surviving intratesticular hmNPCs was confirmed by nestin staining (Fig. 5H–5K) and FISH‐hChr17 (Fig. 5L–5O).

## Discussion

Although there is increasing knowledge about human fetal brain‐derived neural progenitor cells, standardized data on gene profiling of fetal tissue and primary hmNPCs during prolonged proliferation and differentiation are limited 11, 17, 18, 36, 37. Many authors have raised concerns about early senescence of neural progenitor cells in vitro 15, 38, 39, 40. The aim of the present study was a detailed prospective analysis of three different hmNPC lines using GMP‐compliant techniques, to provide robust preclinical data on growth rate, characterization, gene expression, maintenance of stemness, regional identity, and safety for cell therapeutic use or as an unaltered and stable cellular platform for drug screening.

Chromosome stability and analysis of 84 genes involved in DNA repair revealed no indication for changes up to passage 20, which corresponds to a longstanding propagation period of approximately 1 year. The proliferation capacity was assessed via cell counts; ICC for KI67 and nestin; CD15, CD133, and CD184 FACS; and qPCR for markers of pluripotency and proliferation. Growth curves demonstrated that there was continued exponential growth in cultures derived from donors of 10 to 14 weeks of gestation. Tissue derived from the 14‐week‐old donor seemed to grow most efficiently, but all samples had satisfying growth rates and most importantly did not slow down at later passages. In the up‐scaled GMP‐compliant long‐term cultivation process, the yield per hmNPC line was high enough to generate a biobank with clinical samples for therapeutic purposes. We detected an increase in expression of stem cell markers over time; thus, establishing stemness and proliferation potential are preserved during prolonged proliferation in defined medium conditions. Our data provide novel and surprising evidence that stemness of tissue‐specific hmNPCs derived from primary tissue can be increased without genetic manipulation. The mechanisms by which such genes are upregulated remain obscure. One could argue that at least during the initial five passages, the dying off of primary cells could account for this increase. In addition, cell‐cell interactions could help to stabilize stem cell characteristics. However, we believe that low oxygen conditions and hypoxia‐inducible factor (HIF)‐1α‐dependent pathways play a prominent role. We have previously shown that low oxygen and activation of HIF‐1α are crucial for allowing long‐term proliferation of human and murine midbrain‐derived neural progenitor cells 11, 14, 15, 18. It is now widely accepted that HIF‐1α‐dependent signaling pathways not only are important to promote stemness of neural progenitor cells but also promote dopaminergic differentiation 41, 42, 43. Furthermore, there is evidence for a direct effect of HIF‐1α on WNT signaling 44.

If detailed analyses of primary tissue provide the prerequisite for culturing hmNPCs that harbor predopaminergic cells, one still has to be concerned about the ability of such cells to maintain the potential to differentiate into dopaminergic neurons. In this study, we could clearly demonstrate that hmNPCs remain stable during the first 20 passages in vitro, which corresponds to a longstanding proliferation period of approximately 1 year. Moreover, all changes that we were able to identify pointed toward a significant increase of differentiation potential compared with early passages, despite continued growth. This is in line with reports that functional maturation of cultivated human cells mimics the timing of human brain development. Therefore, functional effects and integration of hmNPCs may only be observed during extended time spans of many months and even years 45, 46.

*OTX2*, *PAX5*, *EN1*, *EN2*, *PITX3*, *TH*, and floorplate markers *FOXA2* and *CORIN* mRNA were detected in primary fetal human midbrain and hmNPCs during long‐term cultivation in GMP‐compliant conditions, in addition to corin and TH protein expression. Other neuronal phenotypes (GABA, glutamate 5‐HT, and ChAT) constituted 2.4%–0.8% of the total population of neurons. We used *TH* as marker gene for late dopaminergic differentiation. TH could be detected in primary tissue as well as hmNPCs during proliferation and differentiation, supporting the notion that our cultures preserved the regional identity of the original midbrain tissue, which consists of neural progenitor cells with floorplate fate. If stemness and the proliferation potential are preserved during prolonged proliferation, one would also expect that differentiation into specific neurons of interest would not be impaired. Accordingly, we were able to show that hmNPCs not only maintain but rather increase their differentiation capacity into TH‐expressing neurons, thus mimicking human brain development. Although TH expression alone may not be sufficient evidence to prove dopaminergic differentiation, we could also show that other midbrain dopaminergic A9 markers, such as corin, were upregulated in parallel. Again, HIF‐1α could play a prominent role.

Electrophysiological recordings of voltage‐gated ion channels and action potentials demonstrated the maturation of functional properties in hmNPCs so far only during 4 weeks of differentiation in vitro 47, 48. In agreement with previous studies investigating KCl‐induced dopamine release of embryonic stem cell‐derived human dopaminergic neurons 49, 50, 51, 52 and in fetal hmNPCs by high‐performance liquid chromatography 53, we established the potency of our hmNPCs by dopamine ELISA, showing significantly elevated dopamine levels of TH‐positive hmNPCs after differentiation, thus suggesting the development of functionally active dopaminergic cells during maturation in vitro.

In vivo, partial reduction of motor deficits in graft recipients was paralleled by recovery of specific dopamine transporter binding (PET imaging). By using PET imaging, we found that grafted hmNPCs cells developed into DA neurons, consistent with postmortem dopaminergic immunocytochemical analysis. Behavioral compensation was significant (∼60%), but not complete. Graft recipients of differentiated hmNPCs showed a slightly better compensation of motor asymmetry, which could point to the fact that hmNPCs would also need more time for differentiation. These results are in line with previous transplantation experiments with unaltered NPC lines 21, 54, 55, 56, 57, showing amelioration rather than complete motor restoration in xenogenic transplantation models over a time period of 12 weeks. Functional recovery in the xenogenic model produces much more variable results, when compared with allo‐ or syngeneic models 21. In addition, factors such as differentiation stage 58, region of the host brain 59, survival time of graft recipients 46, and innervation via axonal outgrowth 60, 61 affect regenerative potential. Thus, transplantation of human cells into the rat or monkey brain may pose additional challenges that may or may not be relevant to allogeneic clinical applications.

Grafted hmNPCs were immunoreactive for the human markers Stem121, MAP1B, GIRK2, and synaptophysin 1. In concert with significantly increased neurite length 12 weeks postgrafting, these data point to integration and synaptic plasticity of transplanted hmNPCs in the graft recipients. The survival and high differentiation rate of hmNPCs in vivo may indicate that the midbrain floorplate fate, which is required for the commitment of dopaminergic neurons from hES and IPS cells 3, 8, 62, is preserved in expanded hmNPCs. Hence, the additional use of small molecules such as glycogen synthase kinase 3β inhibitors, Shh agonists, growth factors, and inhibitors 63, which have to be produced under GLP/GMP compliance when used in a GMP process, may not be required in fetal hmNPCs. Additional experiments using different cell dosages in immunodeficient mice and surrogate models may be important to address the issue of local donor cell integration, axonal outgrowth, and immune response.

One important aspect of cell therapy is safety, because continued proliferation must be excluded to minimize the risk of tumor formation 29, 64. Like other studies 65, our GLP‐compliant safety study (toxicology and safety), conducted in immunodeficient nude mice 28, 29, 30, displayed no indication for toxic effects or teratoma formation during the observation period of 30 weeks. With FISH analysis for chromosome 17, we found few hmNPCs within the testes. Substantial differences between environments of the brain and testis may lead to fewer cells in the testis than expected. Thus, we hypothesize that differences in niche‐preference of cells considerably influence adaptation and growth of the cells, and a relatively low number of NPCs may survive in the testis despite extreme dosages of hmNPC.

## Conclusion

The data presented on hmNPC lines propagated in a GMP‐compliant long‐term cultivation process provide robust preclinical data on authentication of three different hmNPC lines, their midbrain regional identity, and evidence that cell replacement therapy using hmNPCs could be an option for patients with Parkinson's disease.

## Author Contributions

J.M., S.C.S., and J.S.: conception and design, financial support, administrative support, provision of study material or patients, collection and/or assembly of data, data analysis and interpretation, manuscript writing, final approval of manuscript; H.‐S.L.: data analysis and interpretation, final approval of manuscript; J.M.K., Y.‐E.L., B.K., M.‐Y.S., and J.S.K.: collection and/or assembly of data, final approval of manuscript; G.H.: financial support, data analysis and interpretation, final approval of manuscript; F.W.: data analysis and interpretation, manuscript writing, final approval of manuscript; H.‐M.C., S.W.C., and K.Y.C.: financial support, administrative support, final approval of manuscript; K.‐S.K.: conception and design, financial support, administrative support, data analysis and interpretation, manuscript writing, final approval of manuscript.

## Disclosures of Potential Conflicts of Interest

H.‐S.L. has uncompensated employment and stock options. J.S.K. has uncompensated employment, intellectual property rights, consultant advisory role, honoraria, research funding, stock options, and expert testimony. The other authors indicated no potential conflicts of interest.

## Supporting information

Supporting Information
