# Supplementary material for: Preclinical Analysis of Fetal Human Mesencephalic Neural Progenitor Cell Lines: Characterization and Safety In Vitro and In Vivo
Source: Stem Cells Transl Med. 2016 Sep 2;6(2):576–88. doi: 10.5966/sctm.2015-0228 (PMC5442800; doi:10.5966/sctm.2015-0228)
Supplement: Supplementary file 1 — Supporting Information [file SCT3-6-576-s001.pdf]

Supplemental Information – Kim and Schwarz et al.

Supplemental Material and Methods

Immunoblotting

Protein extracts from treated and control expanded or differentiated hmNPCs were prepared by centrifugation and lyses in buffer [10mM HEPES-KOH (pH 7.9), 10mM KCl, 1.5mM MgCl2, 0.1% NP-40], supplemented with protease inhibitor cocktail (Roche). Protein concentration was determined using the ECL method (Pierce). Cell lysates (30$\mu g$) were mixed with sample loading buffer [125mM Tris-HCl (pH6.8), 4% (wt/vol) sodium dodecyl sulfate, 20% (vol/vol) glycerol. 200mM dithiothreitol, 0.04% (wt/vol) bromophenol blue]. Proteins were resolved on a sodium dodecyl sulfate-12% polyacrylamide gel and transferred to a PVDF. Equal loading and transfer was verified after staining of membranes with Ponceau. Membranes were blocked with 5% (wt/vol) skim milk in TBS-T (TBS, 0.1% (vol/vol) Tween 20) for 2 hours at room temperature and subsequently incubated with desired primary antibody diluted in TBS containing 5% skim milk and 0.1% Tween 20 overnight at 4°C with gentle agitation. The antibodies are listed S.Table 5. Membranes were washed 3 times with TBS containing 0.1% Tween20 and incubated with horseradish peroxidase-coupled secondary antibodies for 1h at room temperature. Membranes were subsequently exposed with chemiluminescence Western blot detection reagents (Pierce) for qualitative analysis.

Supplemental Figures

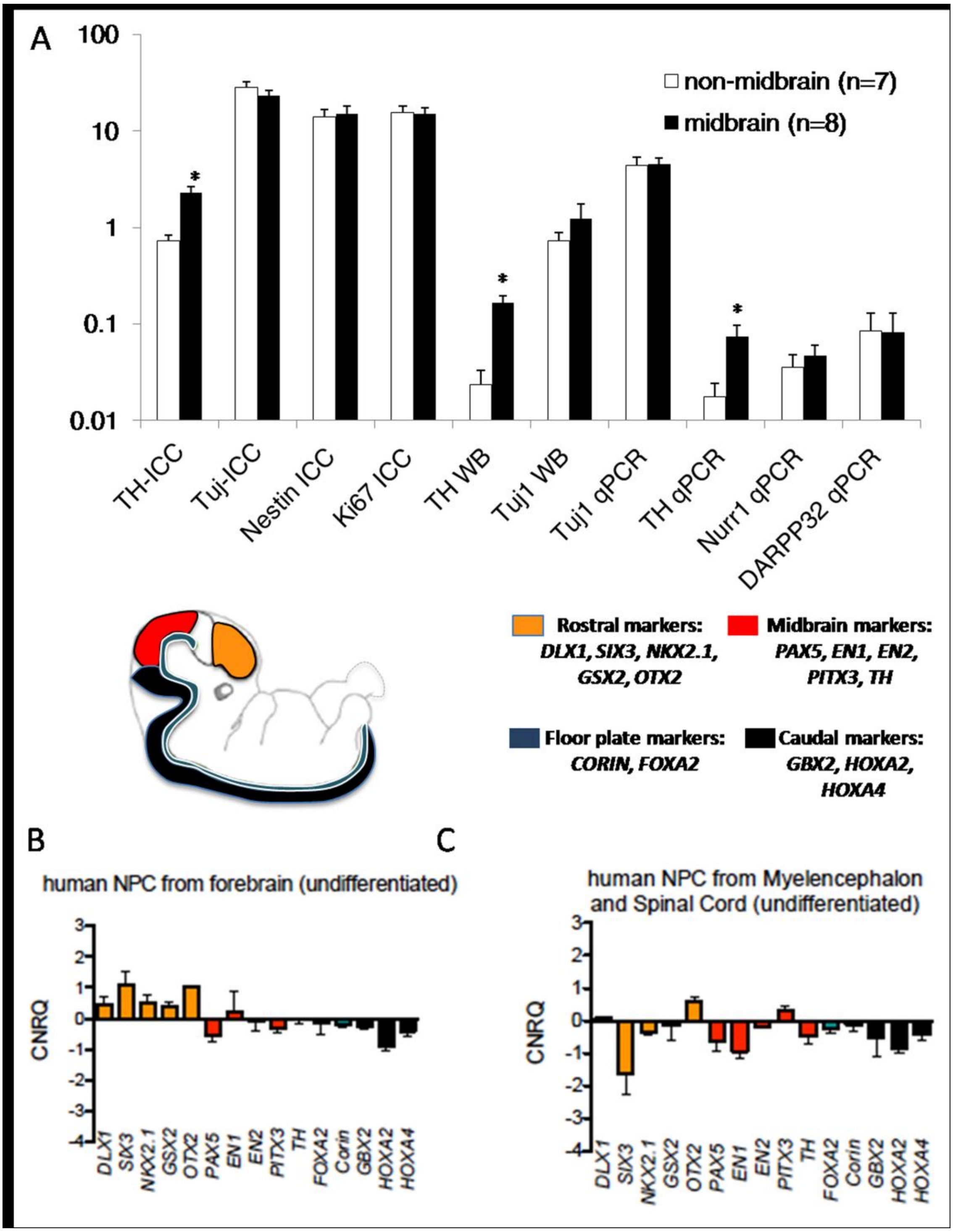

Figure S1 Phenotyping regional identity of hmNPC

(A) Representative ICC, immunoblot and Q-PCR analysis of primary midbrain (n=8) and non-midbrain tissue (n=7) on x-axis; y-axis displays log results on ICC analysis of cell counts (% IR cells compared to DAPI, *p<0.05), immunoblot (TH – or TuJ / Actin ratio, *p<0.005) and Q-PCR ($2^{-\Delta ct}$, *p<0.05).

(B) Gene expression analysis with markers for regional identity in *undifferentiated* hNPC generated from forebrain and

(C) Hindbrain.

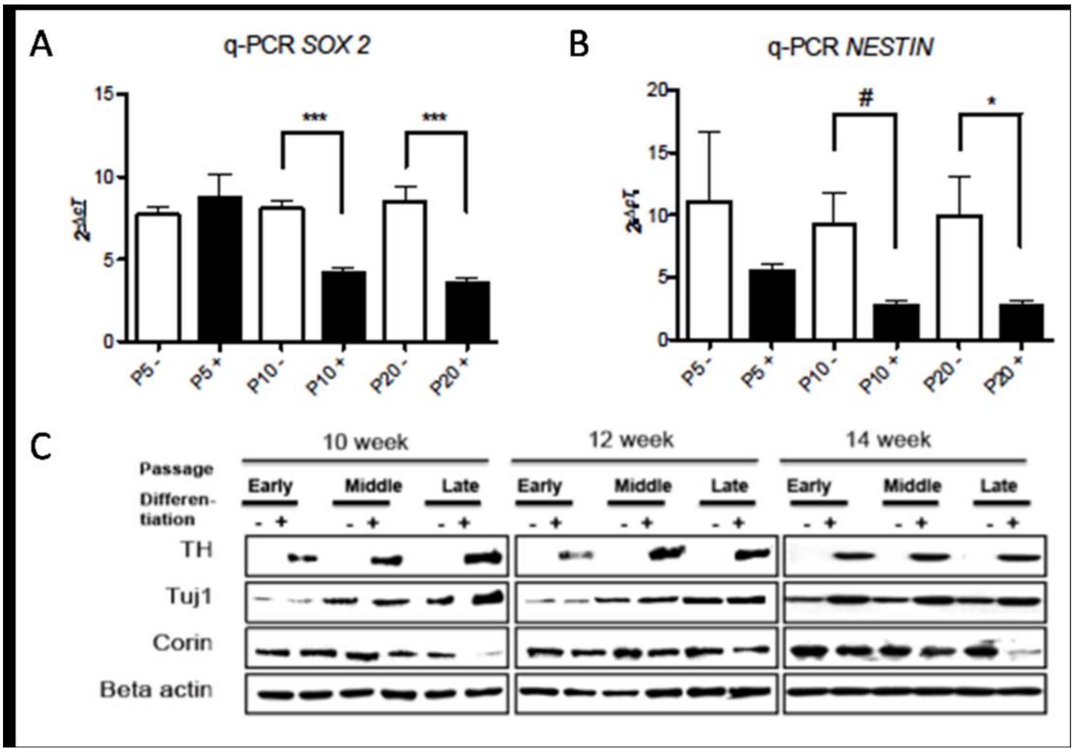


Figure S2 Pluripotency and neuronal characterization of hmNPC

(A) Gene expression analysis for SOX2 (***p<0.005) and

(B) Nestin in hmNPC with (+) and without (-) *differentiation* showing no decrease of SOX2 and Nestin in undifferentiated hmNPC over 20 passages. Markedly lower levels of SOX2 and Nestin were found in differentiated hmNPC, indicating a high proportion of *differentiated* neural cells after 1 week ([#]p< 0.06, *p<0.01).

(C) Immunoblot with TH, TuJ, and Corin antibodies in *undifferentiated* (-) and *differentiated* (+) hmNPC (GW10, 12, 14) in early (P5), middle (P10) and late (P20) passage compared to housekeeping protein Beta Actin.

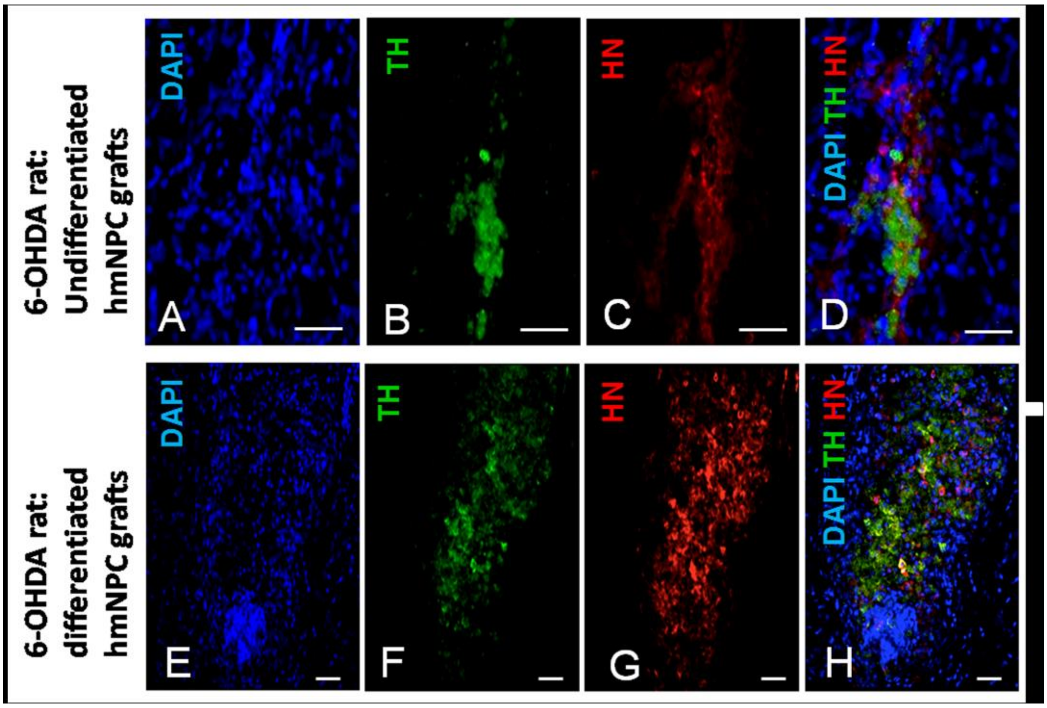


<u>Figure S3 Xenogenic tranplantation</u>

(A) Immunostaining with DAPI of the host striatum grafted with *undifferentiated* hmNPC

(B) Human Nuclei (HN)

(C) TH and the respective

(D) Overlay shows TH immunoreactive cells of human origin in the transplantation area (all scale bars: 50 µm).

(E) Immunostaining with DAPI of the host striatum grafted with *differentiated* hmNPC,

(F) Human Nuclei (HN),

(G) TH and the respective

(H) Overlay confirms that TH immunoreactive cells of human origin are present in the transplantation area 12 weeks postsurgery (all scale bars: 50 µm)

Supplemental Tables

Table S1. Quantitative PCR analysis of *undifferentiated* hmNPC in long-term cultivation: Expression levels at passage 5, 10 and 20 are displayed as $2^{-\Delta ct}$.

| **Undifferentiated hmNPC** | **P5** ($2^{-\Delta ct}$) (mean ± SEM) | **P10** ($2^{-\Delta ct}$) (mean ± SEM) | **P20** ($2^{-\Delta ct}$) (mean ± SEM) |
|---|---|---|---|
| **Sox 2** | 7.69 ± 0.42 | 8.06 ± 0.44 | 9.43 ± 0.93 |
| **Nestin** | 11.11 ± 5.53 | 9.20 ± 2.58 | 9.91 ± 3.23 |
| **PCNA** | 2.70 ± 1.73 | 2.97 ± 1.23 | 2.96 ± 0.55 |
| **Pax 6** | 0.26 ± 0.04 | 0.37 ± 0.04 | 0.26 ± 0.02 |
| **Otx2** | 0.06 ± 0.03 | 0.09 ± 0.01 | 0.03 ± 0.01 |
| **EN1** | 0.07 ± 0.02 | 0.03 ± 0.01 | 0.03 ± 0.01 |
| **TH** | 0.004 ± 0.001 | 0.01 ± 0.005 | 0.021 ± 0.011 |
| **TuJ** | 1.32 ± 0.30 | 1.46 ± 0.12 | 1.74 ± 0.32 |
| **GFAP** | 1.89 ± 1.00 | 0.55 ± 0.34 | 0.29 0.14 |

Table S2. Quantitative PCR analysis of *differentiated* hmNPC in long-term cultivation: Expression levels around passage (P)5, 10 and 20 are displayed as $2^{-\Delta ct}$.

| ***differentiated* hmNPC** | **P5** ($2^{-\Delta ct}$) (mean ± SEM) | **P10** ($2^{-\Delta ct}$) (mean ± SEM) | **P20** ($2^{-\Delta ct}$) (mean ± SEM) |
|---|---|---|---|
| **Sox 2** | 0.30 ± 0.04 | 0.22 ± 0.02 | 0.16 ± 0.009 |
| **Nestin** | 5.53 ± 0.55 | 2.77 ± 0.37 | 2.76 ± 0.29 |
| **PCNA** | 1.43 ± 0.48 | 1.37 ± 0.04 | 1.12 ± 0.12 |
| **Pax 6** | 0.30 ± 0.04 | 0.22 ± 0.02 | 0.16 ± 0.009 |
| **Otx2** | 0.045 ± 0.01 | 0.095 ± 0.004 | 0.035 ± 0.006 |
| **EN1** | 0.034 ± 0.013 | 0.018 ± 0.007 | 0.026 ± 0.009 |
| **TH** | 0.152 ± 0.030 | 0.180 ± 0.038 | 0.230 ± 0.098 |
| **TuJ** | 3.313 ± 1.012 | 6.937 ± 1.715 | 5.620 ± 1.038 |
| **GFAP** | 1.943 ± 0.575 | 0.719 ± 0.301 | 0.347 0.043 |

Table S3. Analysis of *undifferentiated* hmNPC in long-term cultivation: immunoreactive (IR) cells at passage 5, 10 and 20 in relation to total amount of cells (% of DAPI).

| **Undifferentiated hmNPC** | **P5** (%) (mean ± SEM) | **P10** (%) (mean ± SEM) | **P20** (%) (mean ± SEM) |
|---|---|---|---|
| **Ki67** | 20.9 ± 1.6 | 24.3 ± 2.2 | 20.3 ± 1.4 |
| **Sox 2** | 88.2 ± 1.6 | 90.6 ± 1.2 | 91.8 ± 1.3 |
| **Pax 6** | 94.1 ± 1.5 | 93.4 ± 0.9 | 91.3 ± 2.1 |
| **GFAP** | 25.3 ± 5.2 | 23.1 ± 4.9 | 21.7 ± 4.5 |
| **O4** | 1.05 ± 0.3 | 0.8 ± 0.2 | 0.7 ± 0.2 |
| **TuJ** | 12.9 ± 1.1 | 17.9 ± 1.7 | 21.0 ± 2.4 |
| **TH** | 0.4 ± 0.3 | 3.2 ± 1.1 | 4.7± 1.1 |

Table S4 Primer sets of semiquantitative RT-PCR (Q-PCR)

| Template | Primersequence / QuantiTect Order # | Direction |
|---|---|---|
| *c-MYC* | 5'-TCAAGAGGCGAACACACAAC-3' | fwd |
| | 5'-GGCCTTTTCATTGTTTTCCA-3' | rev |
| *CORIN* | 5’-CATATCTCCATCGCCTCAGTTG-3’ | fwd |
| | 5’-GGCAGGAGTCCATGACTGT-3’ | rev |
| *DLX1* | 5’-TGCCAGAAAGTCTCAACAGCC-3’ | fwd |
| | 5’-CGAGTGTAAACAGTGCATGGA-3’ | rev |
| *EN1* | 5’-CGTGGCTTACTCCCCATTTA-3’ | fwd |
| | 5’-TCTCGCTGTCTCTCCCTCTC -3’ | rev |
| *EN2* | 5’-CCTCCTGCTCCTCCTTTCTT -3’ | fwd |
| | 5’-GACGCAGACGATGTATGCAC -3’ | rev |
| *FERD3l* | 5’-GCTGGACTTCGTCGCAGAC-3’ | fwd |
| | 5’-GCCTAATAGGGAGACACCTCTTC-3’ | rev |
| *FOXA1* | 5’-GCAATACTCGCCTTACGGCT-3’ | fwd |
| | 5’-TACACACCTTGGTAGTACGCC-3’ | rev |
| *FOXA2* | 5’-GGAGCAGCTACTATGCAGAGC-3’ | fwd |
| | 5’-CGTGTTCATGCCGTTCATCC-3’ | rev |
| *GATA5* | 5’-CTCAGTTCCTACGCTTCGCAT-3’ | fwd |
| | 5’-GTCGAGGTCAGTGAACAGCA-3’ | rev |
| *GBX2* | 5’-GTTCCCGCCGTCGCTGATGAT-3’ | fwd |
| | 5’-GCCGGTGTAGACGAAATGGCCG -3’ | rev |
| *GFAP* | 5’-AGAGAGGTCAAGCCCAGGAG-3’ | fwd |
| | 5’-GGTCACCCACAACCCCTACT-3’ | rev |
| *GSX2* | 5’-ATGTCGCGCTCCTTCTATGTC-3’ | fwd |
| | 5’-CAAGCGGGATGAAGAAATCCG-3’ | rev |
| *GPBP1* | 5’-ATCATTCGGTCTTCAACCTTCC-3’ | fwd |
| | 5’-ATCCTCAGTTAAGGGAGCACA-3’ | rev |
| *HOXA2* | 5’-CGTCGCTCGCTGAGTGCCTG-3’ | fwd |
| | 5’-TGTCGAGTGTGAAAGCGTCGAGG-3’ | rev |
| *HOXA4* | 5’-ACGCTCTGTTTGTCTGAGCGCC-3’ | fwd |
| | 5’-AGAGGCCGAGGCCGAATTGGA -3’ | rev |
| *KLF4* | 5’-GCCACCCACACTTGTGATTA-3’ | fwd |
| | 5’-CGTCCCAGTCACAGTGGTAA-3’ | rev |
| *LIN28* | 5’-CATCTCCATGATAAACCGAGAGG-3’ | fwd |
| | 5’-GTTACCCGTATTGACTCAAGGC-3’ | rev |
| *NANOG* | 5’-AAGGTCCCGGTCAAGAAACAG-3’ | fwd |
| | 5’-CTTCTGCGTCACACCATTGC-3’ | rev |
| *NESTIN* | 5’-TAAGGTGAAAAGGGGTGTGG-3’ | fwd |
| | 5’-GCAAGAGATTCCCTTTGCAG-3’ | rev |
| *NKX2.1* | 5’-AGGGCGGGGCACAGATTGGA-3’ | fwd |
| | 5’-GCTGGCAGAGTGTGCCCAGA-3’ | rev |

| | | |
|---|---|---|
| *OCT4* | 5’-AGTGAGAGGCAACCTGGAGA-3’ | fwd |
| | 5’-GCCGGTTACAGAACCACACT-3’ | rev |
| *OTX2* | 5’-ACAAGTGGCCAATTCACTCC-3’ | fwd |
| | 5’-GAGGTGGACAAGGGATCTGA -3’ | rev |
| *PAX5* | 5’-CCCCATTGTGACAGGCCGTGAC -3’ | fwd |
| | 5’-TCAGCGTCGGTGCTGAGTAGCT -3’ | rev |
| *PCNA* | 5'-CGGAGTGAAATTTTCTGCAAG-3’ | fwd |
| | 5'-TTCAGGTACCTCAGTGCAAAAG-3’ | rev |
| *POU5F1* | 5’-GTGTTCAGCCAAAAGACCATCT-3’ | fwd |
| | 5’-GGCCTGCATGAGGGTTTCT-3’ | rev |
| *PSMC1* | 5’-CACACTCAGTGCCGGTTAAAA-3’ | fwd |
| | 5’-GTAGACACGATGGCATGATTGT-3’ | rev |
| *RPL22* | 5'-CACGAAGGAGGAGTGACTGG-3’ | fwd |
| | 5'-TGTGGCACACCACTGACATT-3’ | rev |
| *SIX3* | 5’-ACCGGCCTCACTCCCACACA -3’ | fwd |
| | 5’-CGCTCGGTCCAATGGCCTGG -3’ | rev |
| *SOX2* | 5'-AGTCTCCAAGCGACGAAAAA-3’ | fwd |
| | 5'-GCAAGAAGCCTCTCCTTGAA-3’ | rev |
| *SOX17* | 5’-GTGGACCGCACGGAATTTG-3’ | fwd |
| | 5’-GGAGATTCACACCGGAGTCA-3’ | rev |
| *TBP* | 5’-AAAGAACGCTGTACTCAGTGTG-3’ | fwd |
| | 5’-CCCCGGTTGAGGGCTTTTA-3’ | rev |
| *TH* | 5'-AGCCCTACCAAGACCAGACG-3’ | fwd |
| | 5'-GCGTGTACGGGTCGAACTT-3’ | rev |
| *TUBB3* | 5'-GGGCCTTTGGACATCTCTTC-3’ | fwd |
| | 5'-CCTCCGTGTAGTGACC CTTG-3' | rev |
| *UBQLN1* | 5’-TGCAGGTCTGAGTAGCTTGG-3’ | fwd |
| | 5’-5’-AACTGTCTCATCAGGTCAGGAT-3’ | rev |

Table S5. Antibodies for ICC, FACS and WB

| Antibodies | Company | Cat. Number | Application | Concentration |
|---|---|---|---|---|
| Actin | Santa-Cruz | sc-81178 | WB | 1:2000 |
| ChAT | Millipore | MAB305 | IF | 1:500 |
| CD 15 | BD Biosciences | 555401 | FACS | 1:1000 |
| CD 133 | Milteny Biotec | 130-080-801 | FACS | 1:1000 |
| CD 184 | BD Biosciences | 555974 | FACS | 1:1000 |
| Corin | Aviva Systems | ARP46745_T100PAB12761 | WB | 1:200 |
| Doublecortin | Cell Systems | 4604 | IF | 1:200 |
| GABA | Sigma-Aldrich | A2052 | IF | 1:500 |
| GFAP | Zymed | 107-0045 | IF | 1:400 |
| Girk2 | Alomone lab | APC-006 | IF | 1:50 |
| Glutamate | Sigma-Aldrich | G6642 | IF | 1:500 |
| 5-HT | Immuno Star | 20080 | IF | 1:500 |
| Human Nuclei clone 235-1 | Millipore | MAB1281 | IF | 1:50 |
| Ki67 | Leica | Ki67-MM1 | IF | 1:500 |
| MAP1B[pThr1265] | Novus Biologicals | NBP1-42827 | IHC | 1:500 |
| MAP1B (N-19) | Santa Cruz | Sc-8970 | IHC | 1:500 |
| Nestin | BD | BD_611659, | IF | 1:500 |
| Nestin | Abcam | 82375 | WB | 1:500 |
| NeuN | Millipore | MAB377 | IF | 1:200 |
| Pax6 | Abcam | Ab5790 | IF, WB | 1:500 |
| O4 | Millipore | MAB345 | IF | 1:100 |
| Sox2 | Acris | AP11920PU-N | IF, WB | 1:200 |

| | | | | |
|---|---|---|---|---|
| STEM121 | Stemcells | AB-121-U-050 | IHC | 1:250 |
| Synaptophysin 1 | SY Systems | 101011 | IHC | 1:200 |
| TH | Pelfreez | P40101 | IF, IHC, WB | 1:500 or 1:250 |
| TH | Santa Cruz | H196 | WB | 1:500 |
| TuJ | Millipore | MAB1637 | IF, WB | 1:500 |
